# VALIDATION OF A NEW WATER-PERFUSED HIGH-RESOLUTION MANOMETRY SYSTEM

**DOI:** 10.1590/0102-672020200004e1557

**Published:** 2021-01-25

**Authors:** Rogério MARIOTTO, Fernando A. M. HERBELLA, Vera Lucia Ângelo ANDRADE, Francisco SCHLOTTMANN, Marco G. PATTI

**Affiliations:** 1 Department of Surgery, Federal University of São Paulo, SP, Brazil; 2 Department of Pathology, UninCor Faculty of Medicine, Vale do Rio Verde University, Belo Horizonte, MG, Brazil; 3 Department of Medicine and Surgery, University of North Carolina, Chapel Hill, North Carolina, USA

**Keywords:** Esophageal manometry, Gastroesophageal reflux disease, Achalasia, Esophageal motility disorders, Lower esophageal sphincter, Esophageal peristalsis, Manometria esofágica, Doença do refluxo gastroesofágico, Acalásia, Dismotilidade esofágica, Esfíncter esofagiano inferior, Aperistalse esofágica

## Abstract

**Background:**

High-resolution manometry is more costly but clinically superior to conventional manometry. Water-perfused systems may decrease costs, but it is unclear if they are as reliable as solid-state systems, and reference values are interchangeable.

**Aim:**

To validate normal values for a new water-perfusion high-resolution manometry system.

**Methods:**

Normative values for a 24-sensors water perfused high-resolution manometry system were validated by studying 225 individuals who underwent high resolution manometry for clinical complaints. Patients were divided in four groups: group 1 - gastroesophageal reflux disease; group 2 - achalasia; group 3 - systemic diseases with possible esophageal manifestation; and group 4 - dysphagia.

**Results:**

In group 1, a hypotonic lower esophageal sphincter was found in 49% of individuals with positive 24 h pH monitoring, and in 28% in pH-negative individuals. In groups 2 and 3, aperistalsis was found in all individuals. In group 4, only one patient (14%) had normal high-resolution manometry.

**Conclusions:**

The normal values determined for this low-cost water-perfused HRM system with unique peristaltic pump and helicoidal sensor distribution are discriminatory of most abnormalities of esophageal motility seen in clinical practice.

## Graphical abstract

[Fig f1a]

**Figure f1a:**
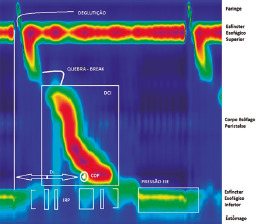
Water-perfused high resolution manometry system

[Fig f1as]

**Figure f1as:**
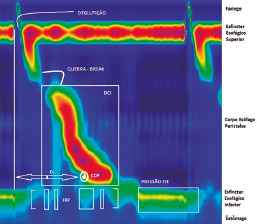
Novo sistema de manometria esofágica de alta resolução por perfusão de água

## RESUMO

**Racional:**

A manometria de alta resolução é mais custosa, porém clinicamente superior à manometria convencional. Sistemas por perfusão de água podem ter custo diminuído, mas não é certo se são tão eficazes quanto aos sistemas de estado sólido e se os valores de referência são intercambiáveis.

**Objetivo:**

Este estudo visa validar valores de normalidade para um novo sistema por perfusão de água.

**Método:**

Valores de normalidade para um sistema de manometria de alta resolução de 24 sensores por perfusão de água foram validados estudando 225 indivíduos submetidos à manometria de alta resolução por queixas clínicas. Pacientes foram divididos em quatro grupos: grupo 1 - doença do refluxo gastroesofágico; grupo 2 - acalasia; grupo 3 - doenças sistêmicas com possível doenças sistêmicas com comprometimento esofágico; e grupo 4 - pacientes com disfagia.

**Resultado:**

No grupo 1, esfíncter esofagiano inferior hipotônico foi encontrado em 49% dos indivíduos com pHmetria positiva e 28% daqueles com pHmetria negativa. Nos grupos 2 e 3, aperistalse foi encontrada em todos indivíduos. No grupo 4, somente um paciente (14%) tinha manometria normal.

**Conclusão:**

Os valores de normalidade definidos para este sistema de manometria de alta resolução por perfusão de água são discriminatórios da maioria das anormalidades da motilidade esofágica vistas na prática clínica.

## INTRODUCTION

High-resolution manometry (HRM) is more intuitive, comfortable and clinically superior as compared to conventional manometry; however, it is very costly^4^ ^,^ ^20^. HRM originated from a water-perfused system^7^ and current parameters were defined based on solid-state systems^14^. Water-perfused systems may decrease costs using cheaper catheters with longer lifespan, but it has limitations on the total number of sensors, jeopardizing the maximum advantage of HRM, namely the high-density of close-spaced sensors. It is unclear if water-perfused systems are as reliable as solid-state systems and reference values may be imported from solid-state systems. 

This study aims to validate normal values in a new water-perfusion HRM system.

## METHODS

The project was approved by local ethics committee. The authors are responsible for the study, no professional or ghost writer was hired. 

### Subjects

Normal values were validated in 225 individuals prospectively studied with specific clinical complaints to encompass a large spectrum of esophageal motility disorders. 

#### Group 1

Individuals under investigation for clinically suspected gastroesophageal reflux disease (GERD, n=156). This group was divided in pH positive (n=103, mean age 45.54±11.78 years, 64 (62%) females), and pH negative (n=53, mean age 43.5±12 years, 38 (72%, females) based on DeMeester score. 

#### Group 2

Patients under evaluation for achalasia. Sample totaled 47 individuals. The mean age was 47.2±16.5 years, 14 males and 33 females.

#### Group 3

Individuals with systemic disease with possible impairment of esophageal motility. The sample totaled eight individuals. The mean age was 52±17.7 years, three males and five females. There were six patients with systemic sclerosis, one with myasthenia gravis and one with clozapine usage.

#### Group 4

Fourteen patients under evaluation for dysphagia who were not included in the prior groups. The sample totaled 14 individuals. The mean age was 55.26±17.2 years, four males and 10 females. There were three patients who underwent a Nissen fundoplication.

### High-resolution manometry

HRM was performed as previously described^22^. The test was performed after 8 h fasting, and discontinuation of medications that could affect esophageal motility. The system was calibrated per manufacturer instructions. After a period for adaptation to the catheter, individuals were instructed to avoid swallowing for a period of 30 s in order to acquire resting parameters; subsequently 10 swallows of 5-ml every 30 s were given to acquire dynamic parameters. All tests were performed and interpreted by a single experienced esophagologist^21^. 

**FIGURE 1:**
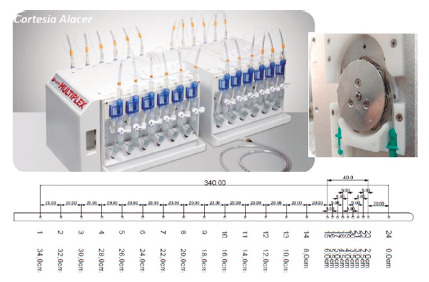
Low-cost water-perfused high-resolution manometry system with unique peristaltic pump (inset) and helicoidal sensor distribution (scheme)

**FIGURE 2:**
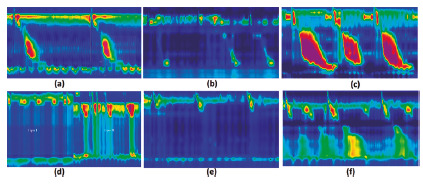
Examples of esophageal motility disorders obtained with a water-perfused high-resolution manometry system: A) normal peristalsis; B) ineffective motility in a patient with gastroesophageal reflux disease; C) jackhammer esophagus in a patient with dysphagia; D) achalasia; E) absent peristalsis in a patient with connective tissue disease; F) distal spasm

The HRM system consisted of a 24-channel water-perfused catheter (Multiplex, Alacer Biomedica, São Paulo, Brazil). The reusable polyvinyl chloride (PVC) catheter had channels in different configuration for the analysis of the pharynx, the esophageal body and the lower esophageal sphincter (LES). Fourteen unilateral channels 2 cm a part (covering 28 cm) were used for the pharynx and esophageal body, while nine spiral channels at 5 mm intervals and angled 120° were used for the LES area (covering 4 cm). One channel was used to record gastric pressure (34 cm in total). Water-perfusion was provided by an original patented controlled peristaltic pump (Figures 1 and 2).

#### Normal values

Normal values for this new water-perfused HRM system were defined previously on 32 healthy volunteers^22^.

### Manometric parameters

Manometric parameters evaluated were those standardized by the International High-Resolution Manometry Working Group in 2015, the Chicago classification 3.0^9^, with the addition of upper esophageal sphincter (UES) basal and relaxation pressures, and LES basal pressure, total and abdominal lengths that were part of the Chicago classification^11^. Data was obtained based on automated analysis by the dedicated software (Esofagica v.1492. Alacer Biomedica, São Paulo, Brazil).

### pH monitoring test

Esophageal ambulatory pH monitoring (AL3, Alacer Biomedica, São Paulo, Brazil) was performed in all patients in group 1 after discontinuation of acid reducing medications. Patients were considered pH positive if the composite DeMeester score was higher than 14.7. 

## RESULTS

### Group 1 - GERD

Manometric parameters are shown in Table 1. In patients with GERD, confirmed by pH monitoring test, 51 patients (49%) had a hypotonic LES and 21 (20%) had ineffective esophageal motility (IEM). Among individuals with normal pH monitoring test, hypotonic LES, IEM and distal esophageal spasm (DES) were present in 15 (28%), five (9%) and six (11%) individuals, respectively.

**TABLE 1:** Manometric parameters and diagnosis in individuals under investigation for clinically suspected gastroesophageal reflux disease (GERD, n=156)

Parameter		Average +/-Standard deviation [range]	Median (IQ)		
LES	IRP	2.66 +/- 6.14	2.10		
	LES BP	11.69 +/- 10.40	9.6		
Body	DCI	869.89 +/-769.47	645.05		
	DL	7.32 +/- 1.86	7.30		
	Break	2.54 +/- 2.34	2.35		
UES	UES BP	71.31 +/- 54.25	49.60		
Manometric diagnostics				
	Normal	Hypotonic LES	IEM	DES
Group 1A	20 (19%)	51 (49%)	21 (20%)	26 (25%)
Group 1B	26 (49%)	15 (28%)	5 (9%)	6 (11%)

IRP=integrated relaxation pressure; LES=lower esophageal sphincter; UES=upper esophageal sphincter; DCI=distal contractile integral; DL=distal latency; LES BP=lower esophageal sphincter basal pressure; UES BP=upper esophageal sphincter basal pressure; DES=distal esophageal spasm; IEM=ineffective esophageal motility

### Group 2 - achalasia

Manometric parameters and are shown in Table 2. All individuals had aperistalsis. In nine (19%) of the cases it was not possible to evaluate the LES. Incomplete relaxation was present in 24 (63%) of the cases when the LES was studied. Sixteen (34%) individuals were classified as achalasia type I, 31 (66%) type II, while no patient had type III achalasia.

**TABLE 2:** Manometric parameters, findings and types in individuals under evaluation for achalasia (n=47)

Parameter		Average +/-Standard deviation [range]	Median (IQ)	Observation
LES	IRP	17.87 +/- 11.51	18.85	19 % of defective LES
	LES BP	32 +/- 20.15	29	
Body	Peristalsis (%)	0	0	100% of aperistalsis
UES	UES BP	66.64 +/- 32	58.80	
Manometric diagnostics				
Type I Achalasia		16 (34%)	Type II Achalasia	31 (66%)

IRP=integrated relaxation pressure; LES=lower esophageal sphincter; UES=upper esophageal sphincter; DCI=distal contractile integral; DL=distal latency; LES BP=lower esophageal sphincter basal pressure; UES BP=upper esophageal sphincter basal pressure

### Group 3 - systemic diseases

Manometric parameters are shown in Table 3. All individuals had absent peristalsis.

**TABLE 3:** Manometric parameters and findings in individuals with systemic disease with possible impairment of esophageal motility (n=8)

Parameter		Average +/-Standard deviation [range]	Median (IQ)	Observation
LES	IRP	2.12 +/- 3.15	1.9	50% of defective LES
	LES BP	13.78 +/- 13.81	7.55	
Body	DCI	19.41 +/- 39.08	1	
	DL	0	0	
	Peristalsis (%)	0.01 +/- 0.03	0	100% of aperistalsis
UES	UES BP	54.10 +/- 24.57	50.80	

UES BP - Upper Esophageal Sphincter Basal Pressure; IRP=integrated relaxation pressure; LES=lower esophageal sphincter; UES=upper esophageal sphincter; DCI=distal contractile integral; DL=distal latency; LES BP=lower esophageal sphincter basal pressure; UES BP=upper esophageal sphincter basal pressure

### Group 4 - dysphagia

Manometric parameters are shown in Table 4. All patients had abnormal manometry.

**TABLE 4:** Manometric parameters, findings and diagnoses in individuals with dysphagia (n=14)

Parameter		Average +/-Standard deviation [range]	Median (IQ)		Observation	
LES	IRP	3.89 +/- 6.91	1.85		14 % of defective LES	
	LES BP	27.61 +/- 23.07	23.05			
Body	DCI	4597.35 +/- 4994.27	3260.70		21 % of aperistalsis	
	DL	6.13 +/- 4.34	7.3			
	Break	1.26 +/- 1.81	0.1			
UES	UES BP	62.17 +/- 49.62	35.95			
Manometric diagnostics					
Normal	Jackhammer	absent of contractility	DES	EGJ junction outflow obstruction	Not classifiable
1 (14%)	7 (50%)	3 (21%)	1 (14%)	1 (14%)	1 (14%)

IRP=integrated relaxation pressure; LES=lower esophageal sphincter; UES=upper esophageal sphincter; DCI=distal contractile integral; DL=distal latency; LES BP=lower esophageal sphincter basal pressure; UES BP=upper esophageal sphincter basal pressure; DES=distal esophageal spasm; EGJ=esophagogastric junction

## DISCUSSION

### Normative values

Very interestingly, the same normal values^18^ were adopted by most authors irrespective of the used system. The same occurred at the beginning of the adoption of the HRM in clinical practice. Later, however, most authors realized that manometry systems are different and normative values must be defined for each type of equipment. There are different water-perfused systems available in which normal values were defined (Table 5)^2^ ^,^ ^3^ ^,^ ^9^ ^,^ ^24^. They clearly differ from solid state systems as they are associated with longer time variables and lower amplitudes due to the physical characteristics of the flow sensors. Normal values can always be obtained by recruiting and studying health volunteers; however, validation of the attained values must be always desirable in order to prove clinical application of this data. Our results show that solid-state reference values are not compatible with water perfused systems and that the reference values we studied for this specific system are adequate and sensitive in order to discriminate most motility disorders. 

**TABLE 5:** Normal values for high-resolution water-perfusion esophageal manometry systems compared to the Chicago consensus

	Current study values ^22^	Tseng et al ^24^	Kessing et al ^10^	Burgos Santamaria et al ^2^	Capovilla et al ^3^	Chicago 3.0 ^9^
Number of volunteers	32	66	50	16	20	-----
sensors	24	22	36	22	24	-----
UES BP - mmHg	16.7 -184.37	NA	NA	NA	NA	NA
UES RP- mmHg	- 20.72 - + 5.95	NA	NA	NA	NA	NA
DCI - mmHg.s.cm	83-3837	99-2186	142-3.674	285-2.280	557-1.726	450 - 8000
DL - s	> 6.20	> 6.20	> 6.20	> 6.10	> 7.00	> 4.50
les BP- mmHg	5 - 37	8.70-46.50	< 18.80	< 54	NA	NA
IRP - mmHg	< 16	< 20	< 29.8	< 20	< 8.80	< 15
BREAK - cm	< 7	< 13.40	NA	NA	NA	<3

IRP=integrated relaxation pressure; LES=lower esophageal sphincter; UES=upper esophageal sphincter; DCI=distal contractile integral; DL=distal latency; LES BP=lower esophageal sphincter basal pressure; UES BP=upper esophageal sphincter basal pressure; UES RP=upper esophageal sphincter resting pressure; NA=not achieved CM=centimeters; MMHG=millimeters of mercury.

### Group 1 - GERD

GERD pathophysiology is certainly multifactorial^13^ but a defective LES is present in 50-70% of individuals with abnormal pH monitoring^1^ ^,^ ^5^ ^,^ ^15^ ^,^ ^27^. The rate of defective LES is within these limits in our study. Esophageal body hypomotility is also frequently found in GERD patients. Based on the current classification^9^ the rate of IEM in GERD ranges from 38-50%^8^ ^,^ ^23^. Our rate is lower than in other published studies; however, in negative pH patients the rate is lower, consistent with other studies that show higher acid exposure in patients with IEM^17^. The rate of defective LES was also lower. 

### Group 2 - achalasia

The Chicago classification defines achalasia based on aperistalsis and impaired LES relaxation, and classifies the disease based on esophageal pressurization^9^. In our series, aperistalsis was consistently found in all patients that had untreated achalasia based on symptoms, endoscopic and radiologic evaluation. LES relaxation was, however, normal in 25% of the cases. This number is similar when a solid-state system is used^25^. Although this phenomenon was also found in idiopathic achalasia, it is more common in Chagas´ disease patients that comprised the majority of patients in our series^6^ ^,^ ^25^. For the same reason, achalasia Type III was not diagnosed as it is probably not found in Chagas´ disease esophagopathy^26^. 

### Group 3 - systemic diseases

Esophageal dysmotility when present in patients with connective tissue diseases is usually manifested by absent peristalsis^16^. All patients who underwent HRM had absent peristalsis in our series. However, they might represent biased referrals since they were all very symptomatic. Clozapine usage and myasthenia gravis also be associated with absent peristalsis as seen in our cases^12^ ^,^ ^19^. 

### Group 4 - dysphagia

Esophageal hypermotility and hypomotility may be both causes for functional dysphagia^30^. Both types of motility were found in our series. Esophagogastric junction outflow obstruction is a common cause of dysphagia after a Nissen fundoplication^28^. This diagnosis was found in 33% of the patients evaluated in this series as it is a common cause of postoperative dysphagia^29^. 

## CONCLUSIONS

We studied a water-perfused with permanent catheters HRM system with unique peristaltic pump and helicoidal sensor distribution. It is a low-cost (US$ 20,000) alternative do solid state system (US$ 60,000). The normal values determined for this system were discriminatory of most abnormalities in esophageal motility seem in clinical practice. 
